# Biostimulant-Mediated Enhancement of Quinoa Tolerance to NaCl-Induced Salinity Stress Through Foliar Application of Ascorbic Acid, Saponins and Quinoa Seed Extract

**DOI:** 10.3390/plants15192961

**Published:** 2026-09-28

**Authors:** Rim Ben Youssef, Rahma Goussi, Chiraz Friji, Hatem Ben Jouira, Arafet Manaa

**Affiliations:** Laboratory of Extremophile Plants, Centre of Biotechnology of Borj Cedria, B.P. 901, Hammam-Lif 2050, Tunisia; rim.benyoussef@hotmail.com (R.B.Y.); rahma.goussi@cbbc.rnrt.tn (R.G.); chirazderbali12@gmail.com (C.F.); hatem.benjouira@cbbc.rnrt.tn (H.B.J.)

**Keywords:** quinoa, biostimulant, saponins, quinoa seed extract, ascorbic acid, foliar application, photosynthesis, primary and secondary metabolites

## Abstract

Quinoa (*Chenopodium quinoa* Willd.) is widely recognized for its relatively high tolerance to salinity, but substantial genotypic variation exists, with some varieties remaining sensitive to severe salt stress. Valorizing quinoa biomass and processing-derived resources also offers opportunities for developing zero-waste value chains and producing value-added food and non-food products. This study evaluated foliar applications of saponins (SAP, 0.15%), quinoa seed extract (QSE, 3%), and ascorbic acid (AsA, 0.2 mM) to improve the physiological and biochemical performance of quinoa under salinity (300 mM NaCl). Salinity markedly reduced biomass, photosynthetic pigment content, photosystem I and photosystem II efficiency, and protein levels, while increasing oxidative stress, osmolytes, and secondary metabolites. Foliar treatments substantially alleviated these effects, although responses varied among biostimulants. AsA showed the strongest growth-promoting effect, increasing total dry weight by 115% compared with salt-stressed plants. SAP particularly improved tissue water status, reduced lipid peroxidation, and stimulated osmolyte and antioxidant accumulation, whereas QSE markedly enhanced photosystem II performance. SAP and QSE also promoted biochemical acclimation by increasing levels of proline, soluble sugars, phenolics, flavonoids, tannins, and saponins while reducing malondialdehyde accumulation. Overall, AsA, SAP, and QSE enhanced the resilience of salt-sensitive quinoa under severe salinity, highlighting a potential zero-waste strategy in which quinoa-derived resources, particularly saponins and quinoa seed extract, can be valorized as foliar biostimulants to support sustainable quinoa production in salt-affected environments.

## 1. Introduction

Salinity is one of the major abiotic constraints limiting plant growth and development, as excessive accumulation of soluble salts in the soil solution lowers osmotic potential, impairs water uptake, and triggers a physiological drought response despite the presence of adequate soil moisture [1]. This early osmotic imbalance is subsequently followed by ionic toxicity caused mainly by excessive accumulation of Na^+^ and Cl^−^ ions, which disrupts mineral homeostasis and cellular functions [2]. These combined effects trigger extensive alterations in key physiological and biochemical processes, including nutrient acquisition, respiration, osmolyte accumulation, enzyme activities, photosynthetic efficiency, carbon metabolism, and antioxidant defense systems, ultimately leading to reduced plant growth and productivity [3]. Specifically, salinity stress impairs plant growth by disrupting gas exchange processes and altering the photosynthetic apparatus. Excessive salt accumulation negatively affects the functioning of photosystem I (PSII) and photosystem II (PSI), leading to reduced electron transport efficiency, impaired energy transfer, and an imbalance in energy distribution within the photosynthetic machinery [4]. Moreover, salt-induced oxidative stress can cause damage to both the donor and acceptor sides of PSII, resulting in reduced photochemical performance and decreased photosynthetic capacity [5]. At the same time, salt-induced metabolic disturbances trigger excessive production of reactive oxygen species (ROS), leading to oxidative imbalance and disruption of cellular homeostasis [6,7].

Environmental stress such as salinity is well recognized for stimulating the production of secondary metabolites with potent antioxidant properties, enabling plants to mitigate the excessive accumulation of ROS. Among these metabolites, phenolic compounds play a central role, and a strong positive correlation between antioxidant activity and total phenolic content has been consistently reported across plant species [8]. Besides phenolics, other secondary metabolites, including flavonoids, tannins, anthocyanins, and phenolic acids, make substantial contributions to the antioxidant defense system. In particular, total phenolic content (TPC) and total flavonoid content (TFC), which function as essential biochemical buffers against ROS-induced oxidative damage, are commonly enhanced in response to elevated Na^+^ concentrations under salt stress [9]. Likewise, tannins, which contribute to cell wall reinforcement and protein stabilization, exhibit stress-dependent changes in accumulation, thereby improving cellular protection against the detrimental effects of salinity [10]. Furthermore, by preserving the fluid integrity of membranes and offering a chemical defense against infections, saponins, which are abundant in halophytic species like quinoa, serve as multipurpose agents for salt tolerance [11].

In addition to the plant’s inherent biochemical defenses, the exogenous application of biostimulants, nutrients, and plant growth regulators has gained increasing attention as an effective strategy for enhancing salinity tolerance, particularly through foliar application, which overcomes the limitations of soil–root nutrient uptake, especially under environmental stress conditions such as drought and salinity [12]. Foliar feeding has evolved beyond conventional nutrient supplementation to include plant-regulating molecules and biostimulants, such as phytohormones (e.g., salicylic acid, abscisic acid, and gibberellic acid) [13], nanoparticles [14], and plant extracts [15]. According to recent studies, foliar sprays greatly increase plant resilience by stabilizing photosynthetic pigments and increasing antioxidant enzyme activities, which reduces oxidative damage brought on by abiotic stressors [16,17]. Additionally, because it decreases nutrient leaching and improves the accuracy of delivery for micronutrients and biostimulants like ascorbic acid or plant extracts, foliar distribution is thought to be more environmentally friendly than soil treatment [12]. The effectiveness of these approaches, however, is not uniform across crop species, genotypes, or growing environments. Previous studies have shown that exogenous application of antioxidants and other bioactive compounds can improve growth, photosynthetic performance, antioxidant defense, osmotic adjustment, and yield under salinity, but the magnitude of these responses depends on factors such as genotype, salinity intensity, soil or growing conditions, application concentration, and timing [18]. Such variability highlights the importance of evaluating specific biostimulant–crop combinations under defined stress conditions rather than assuming a universal response.

In this regard, the exogenous application of bioactive compounds and plant-derived extracts has emerged as a promising strategy for enhancing plant tolerance to abiotic stress. Among these compounds, ascorbic acid (AsA) is a key non-enzymatic antioxidant and redox buffer that contributes to the maintenance of cellular redox homeostasis under stress conditions. Under salinity, AsA can directly scavenge reactive oxygen species (ROS) and support the activity of antioxidant enzymes, thereby limiting oxidative damage to cellular membranes and photosynthetic components and contributing to the maintenance of photosynthetic efficiency and ion homeostasis [19,20]. Saponins are triterpenoid glycosides naturally occurring in quinoa seeds and seed coats that may influence plant stress responses through effects on membrane properties and cellular signaling [21]. Saponin-based treatments have been associated with enhanced antioxidant capacity and improved physiological stability under abiotic stress, potentially through modulation of stress-related hormonal signaling and activation of antioxidant and defense responses [22]. In addition, quinoa seed extract (QSE), a crude extract derived from whole quinoa seeds, contains a complex mixture of primary and secondary metabolites and bioactive compounds, including saponins, phenolics, and flavonoids, which may act synergistically as biostimulants. These compounds may contribute to stress tolerance by supporting antioxidant defenses, osmotic adjustment, nutrient metabolism, and photosynthetic functioning [23,24]. Therefore, the application of AsA, SAP, and QSE was hypothesized to mitigate salinity-induced oxidative and physiological disturbances through complementary mechanisms involving redox regulation, antioxidant defense, membrane protection, and maintenance of cellular homeostasis.

Recent experimental studies on the foliar application of *Equisetum arvense* extract further illustrate the potential of plant-derived extracts as foliar biostimulants under salinity. Foliar application of *E. arvense* extract improved growth and reduced lipid peroxidation in salt-stressed tomato plants, while also decreasing proline accumulation, suggesting attenuation of salt-induced physiological stress [25]. Similarly, a study conducted on salt-stressed sweet potato grown in salt-affected soil showed that foliar application of saponin in combination with boron improved vegetative growth, photosynthetic rate, and transpiration, with the 0.15% saponin + 20 mg L^−1^ boron treatment producing marked improvements in several traits [26]. These findings support the potential of plant-derived extracts and saponin-based treatments as sustainable approaches for improving plant performance under salinity, while also highlighting the importance of crop-specific responses, environmental conditions, and treatment combinations when evaluating the effectiveness of exogenous bioactive compounds.

Since its domestication in the Andean region, quinoa has expanded beyond its traditional production areas and is now cultivated or evaluated in diverse environments worldwide. This global expansion reflects its nutritional value and its ability to adapt to contrasting climatic and edaphic conditions [27]. Following the declaration of the International Year of Quinoa by the FAO in 2013, quinoa has gained further global recognition as a climate-resilient crop with significant potential to contribute to food security and sustainable agricultural production under increasingly adverse environmental conditions. Previous research conducted across Europe, North Africa, Asia, and other emerging production regions has demonstrated the potential of quinoa to adapt to drought, salinity, low-fertility soils, and other environmental constraints [28]. However, quinoa performance varies considerably among genotypes and growing environments, reflecting differences in physiological and metabolic responses to local environmental conditions [29]. Given this substantial genotypic variation, future research should extend evaluation to a wider range of quinoa genotypes and contrasting agroecological conditions to identify resilient and productive materials and to determine the consistency of their responses to different biostimulant and nutrient-based treatments. Although quinoa possesses an inherent tolerance to several abiotic stresses, including salinity, the ongoing expansion of salt-affected soils requires the development of complementary agronomic strategies to further enhance its productivity and stress resilience. In this context, the valorization of quinoa-derived resources represents an emerging opportunity to integrate stress management with more circular and zero-waste production systems. In particular, quinoa saponins and seed-derived extracts, which can originate from quinoa processing streams and may otherwise have limited-value applications, could be explored as functional biostimulants rather than discarded or underutilized materials. However, their potential, alone or in combination with established biostimulants such as ascorbic acid, to improve quinoa performance under severe salinity remains insufficiently characterized. Evaluating these approaches may therefore contribute to the development of more sustainable strategies for maintaining quinoa productivity and resilience in salt-affected environments. Therefore, the present study aimed to evaluate the effects of foliar application of ascorbic acid (AsA), saponins (SAP), and quinoa seed extract (QSE), applied individually and in combination, on growth, plant water status, photosynthetic performance, including PSI and PSII photochemistry, and primary and secondary metabolism of quinoa under severe salinity stress (300 mM NaCl). By integrating physiological, photosynthetic, biochemical, and phytochemical responses, this study provides insights into the capacity of quinoa-derived resources to function as foliar biostimulants and highlights their potential valorization within a zero-waste approach for improving quinoa resilience to salinity.

## 2. Results

### 2.1. Plant Growth Performance and Water Status

Plant phenotypes under control conditions, 300 mM NaCl stress, and foliar application of the biostimulants ascorbic acid (AsA), saponin (SAP), and quinoa seed extract (QSE), applied either alone or in combination with NaCl, are shown in Figure 1. After two weeks of treatment, plants exposed to 300 mM NaCl exhibited reduced vegetative growth compared with the control. However, no obvious toxicity symptoms, such as petiole necrosis, leaf chlorosis, or leaf abscission, were observed. Salinity mainly reduced plant height, canopy size, and overall vigor. Foliar application of ascorbic acid (AsA), saponin (SAP), and quinoa seed extract (QSE) alleviated the inhibitory effects of NaCl on plant growth. Plants receiving the combined treatments (NaCl + AsA, NaCl + SAP, and NaCl + QSE) displayed larger canopies, greater shoot development, and improved leaf density compared with plants treated with NaCl alone. Among the tested biostimulants, Foliar application with SAP appeared to produce the greatest improvement in shoot growth and plant vigor under salinity stress, followed by AsA and QSE, although quantitative measurements are required to confirm these visual observations.

These visual observations were further supported by biomass production measurements (Figure 2), which more clearly demonstrated the inhibitory effect of salinity and the growth-promoting effects of the biostimulant treatments. Salt stress (300 mM NaCl) significantly reduced plant biomass, with leaf, stem, and root dry weights decreasing by 48%, 37%, and 42%, respectively, compared with the control. Consequently, total plant dry weight declined by 43%, confirming the marked inhibition of plant growth under saline conditions. Foliar application of biostimulants significantly alleviated this negative effect. Under salinity conditions, the NaCl + AsA treatment showed the strongest growth-promoting effect, increasing DW relative to NaCl alone by 120% in leaves, 113% in stems, and 109% in roots, which led to a 115% increase in total DW. Foliar spray with saponin (SAP) also enhanced biomass production, with increases of 79% (leaves), 53% (stems), 78% (roots) and 71% in total DW, whereas QSE induced moderate but consistent improvements (87% in leaves, 29% in stems, 46% in roots and 60% in total DW) compared with NaCl-treated plants.

Regarding plant water status, NaCl treatment markedly affected the water content (WC) of quinoa plants, as evidenced by a significant reduction in WC across leaves, stems, roots, and total plant tissues (Table 1). Under control conditions, quinoa plants maintained high WC values, reaching 90%, 92%, and 94% in leaves, stems, and roots, respectively, with an overall total plant WC of 92%. Foliar application of AsA, SAP and QSE under non-saline conditions did not cause significant changes in tissue hydration compared with untreated control plants, indicating that these treatments did not alter the natural water balance of well-watered plants. However, exposure to 300 mM NaCl alone caused a pronounced decline in water content across all organs, with values decreasing to 81% in leaves, 80.6% in stems, and 82.9% in roots, representing reductions of approximately 11%, 13%, and 12%, respectively, compared with control plants. This reduction reflects the osmotic stress induced by salinity, which limits water uptake and disturbs cellular water homeostasis. However, foliar treatments with AsA, SAP, and QSE significantly improved WC in salt-stressed plants, although the extent of recovery varied among treatments. Under NaCl stress, the application of SAP and AsA was the most effective in restoring WC across different plant tissues. Compared with NaCl-stressed plants, AsA treatment increased WC by 8.5% in leaves, 10% in stems, and 7% in roots. Similarly, SAP application enhanced WC by 8.1%, 10.5%, and 7.4% in leaves, stems, and roots, respectively.

### 2.2. Photosynthetic Pigments Analysis and PAM Measurements

#### 2.2.1. Photosynthetic Pigments

Table 2 shows that NaCl-induced salinity stress significantly altered the photosynthetic pigment composition of quinoa leaves, resulting in a marked reduction in chlorophyll content, whereas foliar application of AsA, SAP, and QSE partially mitigated these adverse effects. Indeed, exposure to 300 mM NaCl caused a substantial decline in chlorophyll accumulation, with Chl*a*, Chl*b*, and total chlorophyll contents decreasing by approximately 75%, 73%, and 75%, respectively, compared with control plants. However, foliar application of the tested biostimulants significantly improved chlorophyll retention under saline conditions, although the magnitude of recovery varied among treatments. Compared with NaCl-stressed plants, the NaCl + AsA treatment showed the strongest restoration of chlorophyll content, increasing Chl*a*, Chl*b*, and total Chl by 50%, 110%, and 73%, respectively. Similarly, the NaCl + SAP treatment enhanced Chl*a*, Chl*b*, and total Chl contents by approximately 25%, 160%, and 77%, respectively, compared with NaCl alone. However, the recovery induced by QSE spraying was comparatively lower than that observed with AsA and SAP, indicating a weaker but still beneficial protective effect on the photosynthetic apparatus.

The NaCl treatment alone significantly increased carotenoid concentration from approximately 10 to 15 µg g^−1^ FW, corresponding to an increase of approximately 53% compared with the control (Table 3). Foliar application of the biostimulants used further enhanced carotenoid accumulation under salt stress, and specifically NaCl + AsA showed the highest carotenoid content (21.58 µg g^−1^ FW), representing a 39% increase compared with NaCl alone, followed by SAP (27% increase) and QSE (20% increase).

The Chl *a*/*b* ratio decreased slightly under NaCl stress, declining from approximately 1.8 in the control plants to 1.6. Foliar application of SAP or QSE under saline conditions resulted in a further reduction in the Chl*a*/*b* ratio, reaching 0.76 and 0.88, respectively. In contrast, the NaCl + AsA treatment maintained a relatively higher Chl *a*/*b* ratio (1.14) compared with the other biostimulant treatments under salinity stress.

#### 2.2.2. Quantum Yields and Energy Conversion in Both PSI and PSII

The quantum yields and energy conversion efficiencies of both photosystems (PSI and PSII) were simultaneously evaluated using a Dual-PAM-100 system (Figure 3). Salt treatment (NaCl) impaired the photochemical efficiency of both photosystems, as indicated by the significant decrease in the quantum yield of PSI, Y(I), and PSII, Y(II), compared with the control (Figure 3a,b). This decline indicates that salt stress interferes with the functionality of the photosynthetic apparatus and limits electron transfer to downstream acceptors. Foliar application of the biostimulants partially alleviated these reductions, with QSE showing the greatest improvement specifically in Y(II), followed by AsA and SAP. This decrease in quantum yield is accompanied by an increase in Y(ND) (quantum yield of non-photochemical energy dissipation in reaction centers due to PSI donor side limitation) (Figure 3c) and by an increase in quantum yield of light-induced nonphotochemical fluorescence quenching, Y(NPQ).

Concurrently, salinity caused a substantial increase in quantum yield of non-light-induced non-photochemical fluorescence quenching, Y(NO), indicating enhanced non-regulated energy dissipation and a risk of photodamage. Consistent with these disturbances, salinity significantly reduced the electron transport rates of PSI [ETR(I)] and PSII [ETR(II)] (Figure 3e,f), indicating impaired electron transport through both photosystems, particularly at higher light intensities.

In contrast, foliar application of AsA, SAP and QSE moderated both donor- and acceptor-side limitations. Treated plants displayed lower Y(ND) values than untreated salt-stressed plants, suggesting improved electron delivery to PSI, while the reduction in Y(NA) indicated more efficient oxidation of PSI acceptors and restoration of redox homeostasis. These improvements demonstrate that the biostimulant treatments enhanced coordination between the two photosystems and promoted smoother electron flow through the photosynthetic electron transport chain.

In addition, NaCl stress increased Y(NO), indicating enhanced non-regulated energy dissipation and a greater risk of photoinhibition and oxidative damage (Figure 3c). In contrast, foliar application of AsA, SAP, and QSE markedly reduced Y(NO), suggesting improved protection of PSII against uncontrolled energy loss. Interestingly, Y(NPQ) was lower in the treated plants than in the untreated salt-stressed plants (Figure 3a). This reduction indicates that treated plants required less regulated thermal dissipation because a greater proportion of the absorbed light energy was utilized for photochemistry, as reflected by the higher Y(II), Y(I), ETR(II), and ETR(I). Thus, the decrease in Y(NPQ) reflects improved photosynthetic efficiency. Similarly, ETR(I) and ETR(II) were markedly enhanced in treated plants, specifically with QSE treatment as compared to NaCl alone, reflecting improved electron transport capacity, greater stability of thylakoid membranes, and stronger photoprotective mechanisms.

### 2.3. Malondialdehyde Content

Salinity stress significantly increased malondialdehyde (MDA) accumulation in both leaves and roots compared with the control, indicating enhanced lipid peroxidation and oxidative membrane damage (Figure 4a). The increase was more pronounced in roots than in leaves. However, foliar application of biostimulant compounds (AsA, SAP, or QSE) under saline conditions significantly alleviated salt-induced lipid peroxidation, resulting in lower MDA levels than those observed under NaCl treatment alone. Among the tested biostimulants, AsA was the most effective, reducing MDA accumulation by 55% in leaves and 50% in roots, followed by SAP (37% and 44%, respectively) and QSE (26% and 27%, respectively).

### 2.4. Proline and Soluble Sugar Contents

The obtained results showed that salinity stress significantly increased proline accumulation in both leaves and roots compared with the control, indicating enhanced osmotic adjustment under saline conditions (Figure 4b). Proline accumulation was higher in leaves than in roots under salt stress. Specifically, NaCl treatment increased proline content by 115% in leaves and 77% in roots relative to the control. Under saline conditions, foliar application of the biostimulants further amplified proline accumulation in an organ-dependent manner compared with NaCl treatment alone. Among the tested biostimulants, AsA induced the highest proline content, followed by QSE and SAP in leaves, whereas in roots the greatest increase was observed with SAP, followed by AsA and QSE. Compared to NaCl-treated plants, AsA increased proline levels by approximately 52% in leaves and 79% in roots, while QSE increased proline by about 29% and 66%, respectively. Foliar application of SAP enhanced proline accumulation by approximately 15% in leaves and 126% in roots.

Results showed that soluble sugar accumulation was consistently higher in leaves than in roots and was significantly enhanced by salinity in both organs compared with the control (Figure 4c). Specifically, NaCl treatment increased soluble sugar content by approximately 52% in leaves and 113% in roots. Under saline conditions, foliar application of the biostimulants further enhanced soluble sugar accumulation, differentially across organs, compared with NaCl treatment alone. The treatment with NaCl + AsA induced the highest soluble sugar content in both leaves and roots, followed by NaCl + SAP and NaCl + QSE in leaves. Compared with the NaCl treatment, NaCl + AsA increased the soluble sugar content by approximately 48% in leaves and 54% in roots. Likewise, the NaCl + SAP treatment enhanced soluble sugar levels by about 40% in leaves and 35% in roots. Similarly, NaCl + QSE promoted sugar accumulation under salinity, although its effect was less pronounced in leaves (27%) than in roots (53%) relative to NaCl-treated plants.

### 2.5. Protein Content

Data analysis showed that salinity stress significantly reduced protein content compared with the control, with decreases of approximately 38% in both organs (Figure 4d). Foliar application of biostimulants significantly alleviated the negative effects of salt stress on protein content. The most pronounced recovery was observed with NaCl + AsA, which restored protein levels by 60% in leaves and 40% in roots compared with NaCl-stressed plants. These findings indicate that biostimulant application mitigated the detrimental effects of salinity on protein metabolism, thereby contributing to the maintenance of cellular metabolic activity under salt stress conditions. Additionally, foliar application of SAP enhanced protein accumulation, resulting in increases of 43% in leaves and 34% in roots, while QSE also induced significant improvements, increasing protein content by 45% in leaves and 28% in roots compared with salt-stressed plants.

### 2.6. Secondary Metabolites and Bioactive Compounds Analysis

#### 2.6.1. TPC and TFC Contents

The accumulation of total phenolic content (TPC) and total flavonoid content (TFC) was significantly enhanced in both leaves and roots under salt stress (300 mM NaCl) compared with control plants (Figure 5a,b). Specifically, NaCl treatment increased TPC by 61% in leaves and 46% in roots. Likewise, TFC increased markedly, with increases of 108% in leaves and 110% in roots relative to the control. Under saline conditions, foliar application of the biostimulants further enhanced the accumulation of these secondary metabolites in both organs compared with plants subjected to NaCl treatment alone. Among the treatments, NaCl + AsA induced the greatest accumulation of both phenolic and flavonoid compounds in leaves. In contrast, roots exhibited a distinct response pattern. SAP application produced the strongest stimulation of flavonoid accumulation, increasing root TFC by 91% compared with NaCl-treated plants, whereas QSE and AsA increased root TFC by 57% and 23%, respectively. Moreover, SAP significantly enhanced both TPC and TFC levels in roots, highlighting its superior organ-specific effectiveness in promoting the accumulation of phenolic compounds under salt stress.

#### 2.6.2. Phytic Acid Content

The effects of salinity and foliar application of biostimulants on phytic acid content are presented in Figure 5c. Phytic acid content in quinoa leaves and roots was moderately affected by salinity stress with a slight reduction, particularly in roots, compared with control plants, suggesting that salinity may influence phytate biosynthesis, mobilization, or storage processes. In contrast, foliar application of ascorbic acid (AsA), saponin (SAP), and quinoa seed extract (QSE) under saline conditions partially or completely restored phytic acid levels.

In leaves, AsA induced the most pronounced response, increasing phytic acid content by 34% compared with NaCl-treated plants alone, whereas QSE and SAP promoted more moderate increases of approximately 14% and 5%, respectively. In roots, a distinct response shape was observed, with SAP exhibiting the strongest stimulatory effect, leading to a substantial 68% increase in phytic acid content relative to salt-stressed plants. Conversely, AsA and QSE resulted in only slight increases of approximately 6%, compared with NaCl-treated plants. These findings indicate that different biostimulants differentially regulate phytic acid accumulation depending on the plant organ, with AsA primarily enhancing foliar responses and SAP having a stronger effect on root phytate accumulation under saline conditions.

#### 2.6.3. Saponin Content

Based on Figure 6a, saponin content in quinoa leaves and roots was affected by salinity stress and modulated by foliar applications of AsA, SAP, and QSE in an organ-dependent manner. Compared with control plants, exposure to 300 mM NaCl significantly increased saponin accumulation by 94% in leaves and 41% in roots, suggesting that salinity stress stimulated the biosynthesis of these secondary metabolites and confirming their potential role in quinoa defense mechanisms under adverse conditions. Foliar application of biostimulants further influenced saponin accumulation under both non-saline and saline conditions, with distinct organ-specific responses.

In leaves, the NaCl + QSE treatment produced the strongest enhancement, increasing saponin content by 61% compared with NaCl-stressed plants alone, followed by NaCl + SAP and NaCl + AsA treatments, which increased saponin levels by 28% and 17%, respectively.

In roots, saponin accumulation was particularly stimulated by SAP application, showing a 120% increase compared with salt stress alone, while QSE also induced a substantial enhancement of 99%. In contrast, AsA exerted a comparatively moderate effect on root saponin content, with only a 15% increase.

#### 2.6.4. Tannin Content

Tannin content in quinoa leaves and roots was significantly affected by salinity stress and modulated by foliar applications of AsA, SAP, and QSE, showing distinct organ-specific responses (Figure 6b). Compared with control plants, exposure to 300 mM NaCl markedly increased tannin accumulation by approximately 150% in leaves and 60% in roots, indicating that salt stress stimulated the production of these phenolic compounds as part of quinoa’s defense strategy against stress-induced oxidative damage. Foliar application of biostimulants under non-saline conditions also modified tannin accumulation. In leaves, AsA induced the strongest enhancement, increasing tannin content by approximately 130% compared with control plants, followed by SAP, which increased tannin levels by about 65%, whereas QSE resulted in a moderate increase of approximately 15%. In roots, the effects of these treatments were less pronounced, with increases ranging between 20 and 40% compared with untreated control plants.

Under saline conditions, the application of biostimulants further amplified tannin accumulation, particularly in leaves. The combined treatment (NaCl + QSE) produced the highest stimulation, with leaf tannins increasing by 40% relative to NaCl alone, followed by NaCl + AsA (15%) and NaCl + SAP (10%). In roots, all biostimulant treatments enhanced tannin levels, with QSE showing the strongest effect, leading to an increase of 31%, followed by SAP (25%) and AsA (15%), compared with salt stress alone.

## 3. Discussion

Quinoa is widely recognized as a facultative halophyte with remarkable salt tolerance, capable of withstanding NaCl concentrations exceeding 600 mM without developing severe toxicity symptoms [30,31]. In agreement with this halophytic characteristic, quinoa plants subjected to 300 mM NaCl in the present study exhibited reduced growth and biomass accumulation but no visible symptoms of salt toxicity, suggesting that growth inhibition represents an adaptive response to osmotic stress rather than severe salt-induced damage. The salt tolerance of quinoa relies on a combination of interconnected physiological, biochemical, and molecular mechanisms, including (i) maintenance of growth under high salinity conditions, (ii) efficient water uptake and regulation of transpiration, (iii) limitation of ionic toxicity through effective Na^+^ sequestration into vacuoles and maintenance of a high K^+^/Na^+^ selectivity, (iv) preservation of photosynthetic activity, (v) restriction of lipid peroxidation and oxidative damage, and (vi) activation of robust antioxidant defense systems to control reactive oxygen species (ROS) accumulation [4,32]. Among these mechanisms, the maintenance of photosynthetic efficiency represents a central component of quinoa salt adaptation, as it ensures energy production and supports metabolic homeostasis under stress conditions [4]. However, the quinoa genotype investigated in the present study exhibited a relatively lower degree of salt tolerance, as evidenced by the disruption of key photosynthetic processes under 300 mM NaCl stress. Salt exposure significantly reduced the effective quantum yields of PSI [Y(I)] and PSII [Y(II)], indicating impaired conversion of absorbed light energy into photochemical energy. Furthermore, NaCl stress affected the regulation of photosynthetic electron transport, inducing limitations at both the donor and acceptor sides of PSI, which suggests an imbalance between electron supply and utilization. These alterations indicate that, although the studied quinoa genotype maintained its halophytic characteristics by avoiding severe toxicity symptoms, its photosynthetic machinery was more susceptible to salinity-induced disturbances, which may partly explain the observed reduction in growth performance.

Interestingly, the principal component analysis (PCA) further supported the distinct physiological and metabolic responses induced by salinity and foliar biostimulant application (Figure 7). The separation of NaCl-treated plants from the control group along PC1 was mainly driven by the positive association of salinity with MDA, proline, soluble sugars, TPC, TFC, tannins, and saponins, highlighting the simultaneous occurrence of oxidative stress, osmotic adjustment, and activation of secondary metabolism under saline conditions. In contrast, control plants were associated with higher biomass, water content, chlorophyll, carotenoids, and protein levels, reflecting better growth and photosynthetic performance under non-stress conditions. These opposite associations between growth-related traits and MDA further support the negative impact of oxidative stress on plant growth and photosynthetic functioning observed under NaCl treatment.

To counteract the detrimental effects of salinity, recent agronomic research has increasingly focused on the use of exogenous biostimulants to enhance plant stress tolerance [33]. Foliar application of fertilizers or bioactive compounds has emerged as a promising and sustainable strategy for alleviating salinity-induced impairments in plant growth and physiological performance [12]. The efficiency of foliar fertilization depends on nutrient mobility within the plant and the absorption mechanisms of above-ground organs [34]. Absorption is influenced by environmental factors (light, temperature, humidity), spray solution characteristics (concentration, pH, surfactants, chelates) [35], and biological factors (species, variety, leaf age, leaf surface, nutritional status, and developmental stage) [36]. In this context, the present study evaluated the effectiveness of three foliar-applied biostimulants, ascorbic acid (AsA), saponins (SAP), and quinoa seed extract (QSE), applied individually or in combination with 300 mM NaCl, in improving the salinity tolerance of quinoa. Particular attention was given to their effects on plant growth, physiological traits, photosynthetic performance of both photosystems (PSI and PSII), oxidative stress responses, and the accumulation of key secondary metabolites.

The obtained results clearly demonstrate that severe salinity markedly impaired biomass accumulation, photosynthetic performance, and metabolic homeostasis, whereas foliar application of ascorbic acid (AsA), saponin (SAP), and quinoa seed extract (QSE) substantially alleviated these adverse effects. The pronounced reduction in dry biomass of leaves, stems, and roots under NaCl treatment confirms that even relatively salt-tolerant species such as quinoa experience significant growth inhibition under severe salinity. This decline in biomass is consistent with previous studies reporting that excessive Na^+^ accumulation disrupts water uptake, disturbs K^+^/Na^+^ homeostasis, impairs nutrient acquisition, and ultimately restricts carbon assimilation [32,33]. These growth limitations are primarily attributed to osmotic stress during the initial phase of salt exposure, which restricts water absorption and cell expansion, followed by ion toxicity resulting from prolonged Na^+^ and Cl^−^ accumulation, leading to metabolic dysfunction and reduced plant growth [2,37].

Significantly, foliar application of the tested biostimulants markedly enhanced biomass accumulation under saline conditions, with ascorbic acid (AsA) producing the greatest improvement compared with saponin (SAP) and quinoa seed extract (QSE). Ascorbic acid is a key component of the ascorbate–glutathione cycle and plays a central role in scavenging reactive oxygen species (ROS), thereby protecting cellular membranes, preserving chloroplast integrity, and sustaining photosynthetic activity under stress conditions [38,39]. Consistent with our findings, a previous study on the salt-sensitive species *Brassica campestris* demonstrated that exogenous foliar application of 1 mM AsA significantly improved growth by enhancing growth performance, maintaining chloroplast ultrastructure, stimulating antioxidant enzyme activities, and reducing oxidative stress, resulting in the substantial improvement in growth and plant water status observed here. Together with the reduction in MDA content, a reliable indicator of lipid peroxidation, this suggests that AsA effectively alleviates oxidative constraints on cellular functions and carbon assimilation. This protective effect was primarily associated with enhanced antioxidant capacity, as evidenced by the increased accumulation of total phenolic content (TPC), total flavonoid content (TFC), and phytic acid, which collectively contribute to the non-enzymatic antioxidant defense system. Supporting our hypothesis, the PCA analysis (Figure 7) revealed a clear shift in the metabolic profile of salt-stressed plants following foliar biostimulant application. The combined treatments (NaCl + AsA, NaCl + SAP, and NaCl + QSE) were separated from untreated NaCl plants, indicating partial restoration of physiological homeostasis. Among them, NaCl + AsA showed the closest association with biomass, water content, proteins, and photosynthetic pigments, consistent with the stronger recovery of growth, water status, and photosynthetic performance observed following AsA application. In addition, AsA likely preserved photosystem integrity and improved photosynthetic performance by limiting oxidative damage to photosynthetic membranes, as reflected by the restoration of photochemical efficiency and electron transport capacity, with higher Y(I), Y(II), ETR(I), and ETR(II), together with reduced Y(NA) and Y(NO), indicating improved regulation of energy partitioning between photochemical and non-photochemical processes.

The use of foliar applications of SAP and QSE in the present study aligns with sustainable strategies aimed at establishing zero-waste quinoa value chains by developing valorization options for additional non-food applications (e.g., quinoa cosmetics, fibers, feed source, bioinsecticides, feed, soil-enhancing biochar, biofertilizer) as well as innovative processing technology or services. Our results demonstrated that foliar application of SAP and QSE significantly improved plant growth and photosynthetic performance under saline conditions, while also enhancing key metabolic responses, including osmolyte accumulation (proline and soluble sugars) and non-enzymatic antioxidant compounds. However, these beneficial effects were generally less pronounced than those observed with AsA application. It is well established that saponins and plant-derived extracts can act as biostimulants by modulating membrane stability, signaling pathways, and secondary metabolism, thereby enhancing plant tolerance to environmental stresses [21]. Previous studies have demonstrated that a saponin-rich fraction from *Yucca schidigera*, one of the main sources of steroidal saponins, can be proposed as a potential biofertilizer to stimulate plant growth and improve crop quality and productivity in wheat (*Triticum aestivum*) [40]. Moreover, the application of saponin-based treatments, either through seed priming or added to the growth medium, enhanced shoot and root biomass production and reduced oxidative stress markers (MDA and H_2_O_2_ levels) in the model plant *Arabidopsis thaliana* under salt stress. These beneficial effects were associated with improved germination and seedling growth, likely through the regulation of ROS homeostasis and the modulation of stress-responsive gene expression [21].

Similar to AsA, foliar application of SAP and QSE significantly mitigated the detrimental effects of salinity on PSI and PSII photochemical performance and electron transport capacity. Under 300 mM NaCl, plants treated with SAP or QSE exhibited a partial recovery of the effective quantum yield of PSII photochemistry [Y(II)], accompanied by a marked reduction in the quantum yield of non-regulated energy dissipation [Y(NO)], reaching values close to those of the control plants. These results indicate that both biostimulants improved the efficiency of light energy utilization while limiting passive energy losses associated with photoinhibition and photodamage under salt stress. The improvement in Y(II) together with the reduction in Y(NO) suggests that SAP and QSE enhanced the functional integrity of the photosynthetic apparatus, likely by preserving PSII reaction center activity, maintaining electron transport efficiency, and strengthening antioxidant defenses that protect the photosystems against salt-induced oxidative damage.

The improvement of photochemical activity observed with plant-derived extracts such as SAP and QSE may be attributed to their high content of bioactive compounds, including phenolics, flavonoids, and tannins, which enhance antioxidant defense systems, stabilize thylakoid membranes, and maintain redox balance under stress conditions [41]. Aligned with our findings, Moringa oleifera seed water extract has been reported to possess high phytochemical content and strong antioxidant activity [42]. The extract contained a high concentration of total phenolic compounds (90.97 ± 0.13 mg gallic acid equivalents g^−1^) and exhibited potent free radical scavenging capacity against DPPH (EC_50_ = 36.89 ± 0.15 μg mL^−1^), ABTS (EC_50_ = 13.20 ± 0.05 μg mL^−1^), and nitric oxide (EC_50_ = 217.95 ± 0.33 μg mL^−1^) radicals. These findings support the role of phenolic-rich plant extracts as effective natural antioxidants capable of enhancing antioxidant defenses under stress conditions. For quinoa, several studies have demonstrated that its seeds are an excellent source of phenolic compounds, containing substantial amounts of both free and bound phenolic fractions, which contribute to their remarkable antioxidant potential [38,39]. In addition to phenolic acids and flavonoids, quinoa seeds contain a wide range of bioactive phytochemicals that enhance their functional properties [43,44]. Consequently, quinoa seed extracts exhibit strong free radical scavenging activity and high reducing power, often surpassing those of conventional cereal grains, highlighting quinoa as a valuable natural source of antioxidants with promising applications as a plant-based biostimulant [44,45,46]. Based on these antioxidant properties of quinoa seed extract, foliar application of QSE in this study effectively enhanced the antioxidant defense system of salt-stressed quinoa plants, thereby limiting oxidative damage induced by excess reactive oxygen species (ROS). This was evidenced by the marked reduction in MDA content, particularly in roots, following QSE and SAP treatments. This lowest MDA accumulation suggests that these biostimulants efficiently scavenged ROS and preserved membrane integrity, thereby maintaining cellular homeostasis and supporting photosynthetic performance under saline conditions. In parallel, foliar application of QSE and SAP further stimulated the accumulation of proline and soluble sugars, especially in roots, compared with NaCl treatment alone. The enhanced accumulation of these compatible solutes, together with the higher WC values, reflects an improved osmotic adjustment strategy under salinity stress. Proline acts as an important osmoprotectant and ROS scavenger, while also contributing to the stabilization of proteins, membranes, and cellular structures under adverse conditions [47]. Similarly, soluble sugars play multiple roles in osmotic regulation, membrane and protein stabilization, carbon storage, and the maintenance of metabolic activity during stress, thereby supporting cellular homeostasis and recovery [48]. The simultaneous enhancement of osmolyte accumulation likely explains the superior ability of QSE and SAP to alleviate salt-induced osmotic stress, ultimately contributing to improved physiological performance and stress tolerance.

The Pearson correlation results (Figure 8) indicated that proline and soluble sugars, especially in leaves and roots, showed positive correlations with MDA and with TPC, TFC, tannins, and saponins. This suggests that osmolyte accumulation occurred together with the activation of secondary metabolism under salinity conditions. Rather than representing independent responses, these metabolites appear to form part of a coordinated biochemical acclimation strategy. Furthermore, the PCA analysis clearly showed that combined treatments (NaCl + SAP and NaCl + QSE) were more closely associated with proline, soluble sugars, tannins, saponins, TPC, and TFC, supporting their predominant contribution to osmotic adjustment and secondary antioxidant metabolism. The close clustering of TPC, TFC, tannins, and saponins further suggests a coordinated activation of secondary metabolic defenses in response to salinity, particularly following biostimulant application.

Notably, SAP treatment stimulated the accumulation of phenolic compounds and saponins in roots, indicating a pronounced activation of root-associated chemical defenses under salinity. Since SAP was applied exclusively by foliar spraying, the accumulation of these compounds in root tissues is unlikely to result from simple residues remaining on the leaf surface and instead suggests that foliar SAP application induced systemic physiological and metabolic responses that extended beyond the site of application. This systemic response may involve the activation of endogenous pathways associated with the synthesis, accumulation, or redistribution of phenolic compounds and saponins in belowground tissues, thereby contributing to the plant’s overall defense and acclimation to salinity. Nevertheless, the present experimental design does not allow us to definitively distinguish between newly synthesized endogenous compounds and compounds that may have been absorbed and subsequently translocated from the leaves. Further studies using targeted metabolomics, isotopic labeling, or tracer approaches would be required to elucidate the uptake, translocation, and metabolic fate of foliar-applied saponins and to distinguish exogenous compounds from those synthesized or accumulated endogenously.

The moderate increases in phytic acid observed under salinity and following foliar biostimulant application may reflect its dual role in phosphorus storage and metal chelation [49]. Beyond its nutritional and storage functions, phytic acid may also contribute to antioxidant protection through metal chelation, particularly by binding iron and thereby limiting iron-catalyzed hydroxyl radical formation [50]. The contrasting responses between leaves and roots further highlight the organ-dependent regulation of secondary and phosphate metabolism, suggesting that quinoa modulates the allocation and accumulation of defense-related metabolites according to the tissue-specific demands imposed by salinity. Regarding tannin accumulation, salinity appeared to act as an elicitor of tannin biosynthesis in quinoa, with the response being particularly pronounced in leaves. This accumulation was further enhanced by foliar application of QSE, suggesting that these seed-derived bioactive compounds may stimulate phenylpropanoid and other secondary metabolic pathways involved in the synthesis of defense-related compounds. The stronger tannin accumulation observed under the combined treatment (NaCl + QSE) may enhance the antioxidant and ROS-scavenging capacity of quinoa tissues, thereby contributing to the mitigation of salt-induced oxidative damage and strengthening the plant’s adaptive response to salinity.

The timing of salinity exposure is an important factor when considering quinoa resilience. In the present study, plants were first established under non-saline conditions and subsequently exposed to severe NaCl stress, allowing the evaluation of their capacity to acclimate to a relatively swift saline challenge. Following the onset of salinity, plants must rapidly cope with osmotic and ionic constraints that can disturb water relations, photosynthetic activity, and cellular redox homeostasis. Previous work has shown that quinoa can activate physiological and antioxidant mechanisms in response to salinity during early plant development, including adjustments in growth, water relations, and antioxidant capacity [51]. In this context, the beneficial effects of AsA, SAP, and QSE observed in the present study under salinity may be associated with complementary physiological and biochemical mechanisms that facilitate plant acclimation to the imposed stress. AsA, as an important cellular redox buffer, may contribute to ROS detoxification and the maintenance of antioxidant capacity, thereby protecting membrane integrity and the photosynthetic apparatus and supporting photosynthetic performance under salt stress. The positive effects observed following SAP application may be related to its influence on membrane stability and stress-responsive defense mechanisms. Notably, exogenous SAP stimulated endogenous saponin accumulation under salinity, with a particularly pronounced increase in roots (+120% compared with NaCl-stressed plants), suggesting that SAP may act not only as a protective compound but also as an elicitor of the plant’s endogenous saponin-mediated defense response. This enhanced saponin accumulation was accompanied by improved retention of chlorophyll and carotenoids under salt stress, supporting a potential role of saponin-mediated responses in maintaining photosynthetic function and cellular homeostasis. Nevertheless, the present results demonstrate an association between SAP-induced saponin accumulation and improved physiological performance rather than a direct causal relationship.

In the case of QSE, its effects may result from the combined action of multiple naturally occurring compounds present in the crude whole-seed extract, which could contribute to antioxidant defense, osmotic adjustment, nutrient metabolism, and maintenance of photosynthetic activity. Collectively, these responses may help treated plants maintain water status, photosynthetic activity, and metabolic homeostasis during salt exposure, thereby facilitating physiological acclimation to salinity. The differential responses among treatments further suggest that these agents may act through partially complementary mechanisms rather than through a single pathway. However, as specific signaling components and the individual constituents of QSE were not directly characterized in the present study, these mechanisms should be considered potential explanations for the observed physiological and biochemical responses. Further studies integrating targeted metabolomic, hormonal, and molecular analyses would be required to elucidate the underlying signaling pathways.

## 4. Materials and Methods

### 4.1. Plant Material and Growth Conditions

Quinoa (*Chenopodium quinoa* Willd.) variety “Rosa Junín” (PI 596498), originating from the Peruvian Inter-Andean Valleys and provided by the USDA Genebank, was used in this study. This variety is characterized by white seed color, red stripes on the stem, and green inflorescences. Its vegetative cycle ranges from 150 to 160 days. “Rosa Junín” is considered an improved variety with high product homogeneity and quality; however, it is also classified as salt-sensitive [52].

Quinoa seeds were surface-sterilized in 20% (*v*/*v*) sodium hypochlorite for 5 min and rinsed thoroughly with distilled water. The seeds were then sown in a substrate composed of sand and vermiculite (2:1, *v*/*v*) and irrigated with distilled water every two days. Ten-day-old seedlings were transferred into a hydroponic system and acclimated for 10 days under semi-controlled greenhouse conditions (Figure 9). Plants were grown in 5 L containers, with 10 plants per container, and three independent containers were used for each treatment. During the acclimation period, plants were initially supplied with half-strength nutrient solution, followed by full-strength nutrient solution according to Hewitt (1966) [53], containing macronutrients (mM): 0.5 Ca(NO_3_)_2_, 1.25 KNO_3_, 0.5 MgSO_4_, and 0.625 KH_2_PO_4_, and micronutrients (mg L^−1^): 2.8 Fe, 0.55 Mn, 0.06 Zn, 0.06 Cu, 0.32 B, and 0.02 Mo. The experiment was conducted during February and March 2026 in a controlled greenhouse maintained at 24/18 °C and 70/90% relative humidity during the day/night, respectively. The photoperiod increased from 12 h in February to 13 h in March.

### 4.2. Salt Stress Application and Foliar Treatments

At the stage when plants had developed four leaves (BBCH-14 stage, according to Sosa-Zuniga et al. (2017) [54], salinity stress was imposed by gradually adding NaCl to the nutrient solution at a rate of 50 mM per day until a final concentration of 300 mM was reached to avoid osmotic shock. The selection of the salinity level, developmental stage, and stress duration was based on a previous screening study of quinoa genotypes conducted by Derbali et al. (2020) [33], in which plants were exposed to 0, 100, 300, and 500 mM NaCl for two weeks, starting at the BBCH-14 stage. Based on the physiological and biochemical responses observed in that study, 300 mM NaCl was selected as a sufficiently severe and reproducible salinity level to induce clear stress responses while maintaining plant viability. Accordingly, salinity was maintained at 300 mM for two weeks, allowing sufficient time for the establishment of the salinity response and for the foliar treatments (Table 3) with ascorbic acid (AsA, 0.2 mM), saponin (SAP, 0.15%), and quinoa seed extract (QSE, 3%) to exert measurable physiological and biochemical effects.

The quinoa accession used for seed extract preparation was CHEN-470, originally collected from Peru and kindly provided by the USDA Genebank, and was selected based on its high total phenolic content (TPC) and total flavonoid content (TFC). The accession was multiplied under controlled greenhouse conditions at the Centre of Biotechnology of Borj Cedria, Tunisia. For the preparation of the quinoa seed extract (QSE), whole seeds (0.5 g) were thoroughly washed and dried at room temperature. The dried seeds were finely ground into powder and extracted with 5 mL of 70% (*v*/*v*) ethanol, corresponding to a seed-to-solvent ratio of 1:10 (*w*/*v*). The suspension was sonicated for 30 min at 40 °C and subsequently allowed to stand at room temperature before filtration. The solvent was removed using a rotary evaporator, and the resulting extract was further lyophilized. The dried crude extract was weighed and re-dissolved in distilled water to obtain a final QSE concentration of 3% (*w*/*v*) for foliar application. The QSE used in this study was a crude whole-seed extract and was not subjected to further purification or fractionation. Therefore, it represents a complex mixture of compounds naturally extracted from quinoa seeds, including primary and secondary metabolites such as proteins, carbohydrates, lipids, minerals, phenolic compounds, flavonoids, and saponins.

Saponin (SAP) was used as a commercially available product (Saponin, product no. 84510, Sigma-Aldrich, Merck, Germany; CAS No. 8047-15-2), extracted from a Quillaja-derived saponin preparation containing 8–25% sapogenin according to the manufacturer, and was dissolved in 70% ethanol to a final concentration of 0.15% (*w*/*v*), with Tween 20 added at 0.05% (*v*/*v*) as a surfactant. L-ascorbic acid (AsA; product no. PHR1008; Sigma-Aldrich/Supelco, Merck, Germany; CAS No. 50-81-7; MW 176.12 g mol^−1^) was dissolved in 70% ethanol to a final concentration of 0.2 mM, with Tween 20 added at 0.05% (*v*/*v*) as a surfactant. SAP and AsA solutions were thoroughly mixed and freshly prepared before foliar application, while the SAP and AsA products were stored at 4 °C until use.

All spraying treatments (AsA, SAP, and QSE) were performed until complete leaf wetting using a hand atomizer, starting at the onset of salt treatment and repeated periodically (once every 2 days). Applications were performed in a controlled environment during the early morning to minimize air currents that could affect spray deposition. Each treatment was applied separately, with the respective groups of pots physically separated during spraying to prevent contact, drift, or cross-contamination between treatments. Treatments were maintained for two weeks, after which plants were harvested and separated into leaves, stems, and roots for growth measurements. The remaining plant material was pooled, freeze-dried, and stored for subsequent biochemical analyses.

### 4.3. Growth Parameters Determination

At harvest, whole plants were carefully collected, and fresh weight (FW) was immediately recorded for individual organs, including leaves, stems, and roots. Subsequently, the samples were oven-dried at 80 °C for 72 h until constant weight was achieved, allowing the determination of dry weight (DW). The water content (WC) of leaves, stems, and roots was calculated and expressed as a percentage according to the following equation:

WC (%) = [(FW − DW)/(FW)] × 100

### 4.4. Photosynthetic Pigment Analysis

Chlorophyll and carotenoid concentrations were determined spectrophotometrically following the method of Lichtenthaler (1987) [55]. Approximately 200 mg of leaf tissue was placed in a dark glass container containing 5 mL of 80% (*v*/*v*) acetone and incubated in the dark at 4 °C for 72 h. Chlorophyll a (Chl*a*), chlorophyll b (Chl*b*), and total carotenoid contents were then calculated using the following equations:

Chl*a* = 12.25A663 − 2.79A645

Chl*b* = 21.50A645 − 5.10A663

Chl_total_ = 7.15A663 + 18.71A645

Carotenoids = (1000 × A470 × 1.82 [Chla] × 85.02[Chlb])/198

Pigment concentrations were expressed as milligrams per gram of fresh weight (mg g^−1^ FW).

### 4.5. Quantum Yields and Energy Conversion in Both PSI and PSII: Dual-PAM 100 Measurements

The quantum yields and energy conversion efficiencies of both photosystems (PSI and PSII) were simultaneously evaluated using the saturating pulse method, as described by Manaa et al. (2021) [56]. Briefly, measurements were performed with a Dual-PAM 100 system (Heinz Walz GmbH, Germany) equipped with a chlorophyll fluorescence analyzer and a P700 dual-wavelength emitter–detector [57,58]. After dark-adapting quinoa leaves for 30 min, a saturating pulse of white actinic light (AL; pulse width = 300 ms, intensity = 10,000 μmol photons m^−2^ s^−1^) was applied to regulate the maximum fluorescence (Fm) and to obtain the related absorbance parameters. Leaves were then exposed to actinic light of increasing intensities (0, 6, 12, 21, 56, 107, 146, 257, 412, 652, and 1017 μmol photons m^−2^ s^−1^) to measure the quantum yield of photochemical energy conversion in PSII Y(II), the quantum yield of regulated non-photochemical energy dissipation (NPQ), and the quantum yield of non-regulated energy dissipation Y(NO). The following parameters, the quantum yield of photochemical energy conversion in PSI Y(I), the yield of non-photochemical energy dissipation caused by acceptor-side limitation Y(NA), and that caused by donor-side limitation Y(ND), were measured as previously recorded by Baker (2008) [59].

### 4.6. Lipid Peroxidation Assessment

Malondialdehyde (MDA), as an indicator of lipid peroxidation, was quantified by determining 2-thiobarbituric acid-reactive substances (TBARS) following the method of Rao and Sresty (2000) [60]. Fresh ground tissue powder (1 g) was mixed with 10 mL of 0.1% (*w*/*v*) trichloroacetic acid (TCA). The mixture was then centrifuged at 15,000× *g* for 15 min at 4 °C. Subsequently, 2 mL of the supernatant was mixed with 2 mL of thiobarbituric acid (TBA), and the mixture was then incubated at 95 °C for 20 min. The reaction was terminated by rapidly cooling the mixture in an ice bath. The absorbance of the supernatant (532 nm) was measured after centrifugation at 1000× *g* for 10 min at 4 °C, and the value corresponding to non-specific absorbance (600 nm) was subtracted. The extinction coefficient (155 mM^−1^ cm^−1^) was then used to quantify MDA content as the difference between the two absorbance readings.

### 4.7. Proline and Total Soluble Sugars

Proline concentration was determined using the method of Bates et al. (1973) [61]. Briefly, 0.2 g of fresh leaf and root tissue was homogenized in 10 mL of 3% (*w*/*v*) sulfosalicylic acid and incubated at 70 °C for 30 min to extract proline. One milliliter of the extract was mixed with 1 mL of glacial acetic acid and 1 mL of acid ninhydrin reagent and then incubated at 90 °C for 1 h. The reaction was terminated in an ice bath, and the chromophore-containing solution was extracted with toluene. Absorbance of the toluene phase was measured at 520 nm, and the proline concentration was determined using a calibration curve as μmol proline g^−1^ FW.

Total soluble sugars were estimated using the anthrone reagent method, with D-glucose as the standard, following Yemm and Willis (1954) [62]. Approximately 500 mg of fresh tissue was homogenized in 5 mL of 70% ethanol, vortexed for 5 min, and centrifuged at 8000 rpm for 10 min at 15 °C. Then, 200 µL of the supernatant was transferred into glass tubes, followed by the addition of 1 mL of anthrone reagent. The mixture was incubated at 100 °C for 10 min, rapidly cooled on ice, and the absorbance was measured at 625 nm.

### 4.8. Soluble Protein Content

Total soluble protein concentration was assessed following the method described by Bradford (1976) [63]. Fresh ground tissue powder (0.5 g) was mixed with 3 mL of phosphate buffer (KH_2_PO_4_ + K_2_HPO_4_) supplemented with 5% polyvinylpyrrolidone (PVP), 5% glycerol, and 1 mM dithiothreitol (DTT). The homogenates were centrifuged at 14,000× *g* for 15 min at 4 °C, and the resulting supernatants were collected, mixed with Bradford reagent, and then incubated for 5 min. The absorbance was recorded at 595 nm, and the protein concentration was calculated by extrapolation on a standard curve prepared in advance with bovine serum albumin (BSA).

### 4.9. Phytic Acid Content

Phytic acid content was determined according to the method of Latta and Eskin (1980) [64]. The extraction solution (2.4% HCl) was prepared by diluting 5.7 mL of 35.4% HCl to 100 mL with distilled water. The Wade reagent consisted of 0.3% sulfosalicylic acid and 0.03% FeCl_3_·6H_2_O. A standard sodium phytate solution was prepared by dissolving 11.2 mg sodium phytate (Sigma, St. Louis, MO, USA) in 100 mL distilled water. Briefly, 500 mg of quinoa flour was extracted with 10 mL of 2.4% HCl for 18 h under agitation (or 2 h sonication), followed by centrifugation at 10,000 rpm for 20 min at 25 °C. The supernatant was mixed with 1 g NaCl, frozen at −20 °C for 60 min, thawed, and centrifuged again under the same conditions. One milliliter of the filtrate was diluted 25-fold; 3 mL of the diluted extract was then mixed with 1 mL Wade reagent, and absorbance was measured at 500 nm. Phytate concentration was calculated using a standard curve equation.

### 4.10. Secondary Metabolites Quantification

Plant extracts were prepared following the method of Miranda et al. (2023) [65] with slight modifications. Briefly, 0.25 g of quinoa leaves and roots flour was ground and mixed with 5 mL of 70% ethanol and subjected to sonication for 30 min at 40 °C. The mixture was allowed to stand at room temperature, filtered, and the solvent was removed using a rotary evaporator, followed by lyophilization. The resulting crude extract was weighed and re-dissolved in distilled water to a final concentration of 5 mg/mL. This extract was subsequently used for the quantification of secondary metabolites, including saponins, polyphenols, tannins, and flavonoids.

- Total phenolic content (TPC)

Total phenolic content was quantified using the Folin–Ciocalteu method according to Singleton and Rossi (1965) [66]. Briefly, 125 µL of sample was mixed with 500 µL of distilled water and 125 µL of Folin–Ciocalteu reagent (1:9, *v*/*v*). Then, 1.25 mL of sodium carbonate solution was added, and the mixture was incubated in the dark at room temperature for 90 min. Absorbance was measured at 760 nm, and TPC was determined from a gallic acid calibration curve, expressed as mg of gallic acid equivalents per gram of fresh weight.

- Total flavonoid content (TFC)

Total flavonoid content was determined using the aluminum chloride colorimetric method as described by Chang et al. (2002) [67]. In summary, 400 µL of plant extract was mixed with 120 µL of sodium nitrite (5%), followed by the addition of 120 µL of aluminum chloride (10%) and 800 µL of sodium hydroxide (1 M). The absorbance was measured at 510 nm, and TFC was calculated from a quercetin calibration curve, expressed as mg quercetin equivalents per 100 g of sample (mg quercetin/100 g).

- Tannin content

Total tannin content was measured using the vanillin-HCl method according to Broadhurst and Jones (1978) [68]. Briefly, 0.2 mL of plant extract was mixed with 1 mL of vanillin reagent prepared in a mixture of HCl and acetic acid (*v*/*v*). The mixture was incubated in a water bath at 30 °C for 20 min, and absorbance was recorded at 410. Tannin content was determined from a catechin calibration curve and expressed as mg catechin equivalents per 100 g of extract (mg catechin/100 g).

- Saponin quantification

Saponin content was quantified using a colorimetric assay based on the protocols of Patel et al. (2012) [69] and Ncube et al. (2011) [70]. The samples were first mixed with 95% ethanol, followed by the addition of half the sample volume of water, to achieve a final solution concentration of 10 mg/mL. To the obtained extract (125 µL), 125 μL of vanillin 0.8% (*w*/*v*) in ethanol and 1.25 mL of 72% (*v*/*v*) sulfuric acid were added. The mixture was incubated at 60 °C for 10 min, and then cooled in an ice bath. The absorbance was measured at 520 nm, and the saponin content was determined using a calibration curve prepared with saponins of 100% purity as the standard.

### 4.11. Statistical Analyses

The statistical analysis was performed using OriginLab software (Originpro 2020, SR1). The experiment was conducted as one independent experiment, with six biological replicates per treatment. Mean values and standard errors (SE) of the physiological and biochemical parameters were calculated from these six biological replicates. Differences among treatments were assessed using one-way ANOVA, with statistical significance set at *p* < 0.05, followed by Duncan’s multiple range test at the 95% confidence level.

## 5. Conclusions

Despite the inherent salt tolerance of quinoa, severe salinity (300 mM NaCl) substantially impaired plant growth, water status, photosynthetic performance, and metabolic homeostasis. Foliar application of ascorbic acid (AsA), saponins (SAP), and quinoa seed extract (QSE) alleviated several of these salt-induced effects, although their responses varied depending on the physiological and biochemical traits considered. Among the treatments, AsA showed the strongest overall protective effect, particularly by improving biomass accumulation, plant water status, and photosynthetic performance under salinity. SAP and QSE also improved plant performance and promoted biochemical acclimation, including the accumulation of osmoprotective and secondary metabolites. Overall, foliar application of AsA, SAP, and QSE represents a promising approach for enhancing quinoa resilience to salinity. Further validation under field conditions is required to determine their effects on reproductive development, grain yield, seed nutritional quality, and the stability of beneficial metabolites, as well as to optimize their application rates and timing. Beyond improving quinoa performance, future research should also explore how these approaches can contribute to integrated and sustainable quinoa production systems and the development of zero-waste quinoa value chains through the valorization of saponins, seed extracts, biomass, and processing by-products for additional non-food applications, including cosmetics, soil amendments, and biofertilizers.

## Figures and Tables

**Figure 1:**
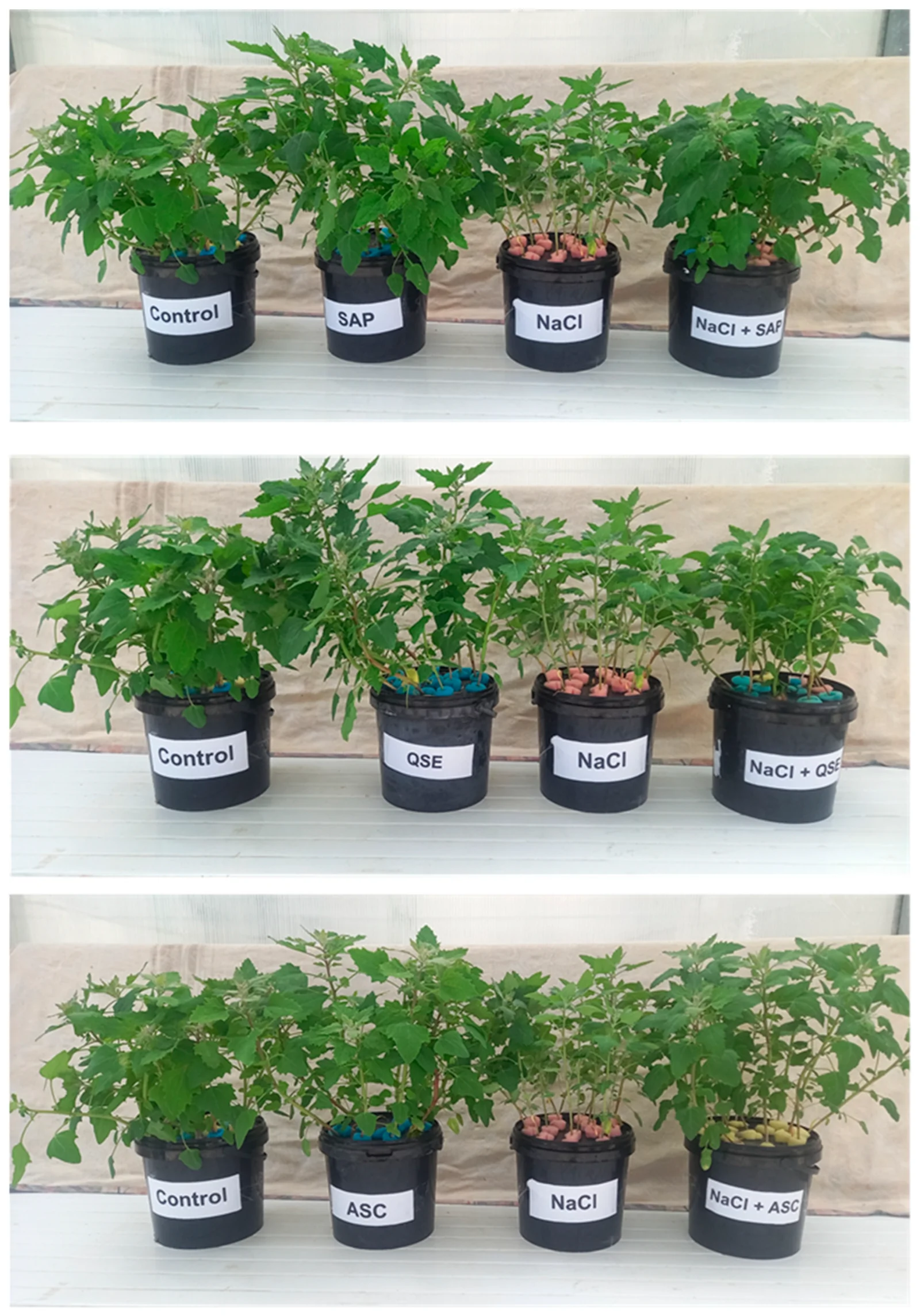
Representative photographs of plants after two weeks of treatment under control conditions, 300 mM NaCl stress, and foliar application of saponin (SAP), quinoa seed extract (QSE), or ascorbic acid (AsA), either alone or combined with NaCl.

**Figure 2:**
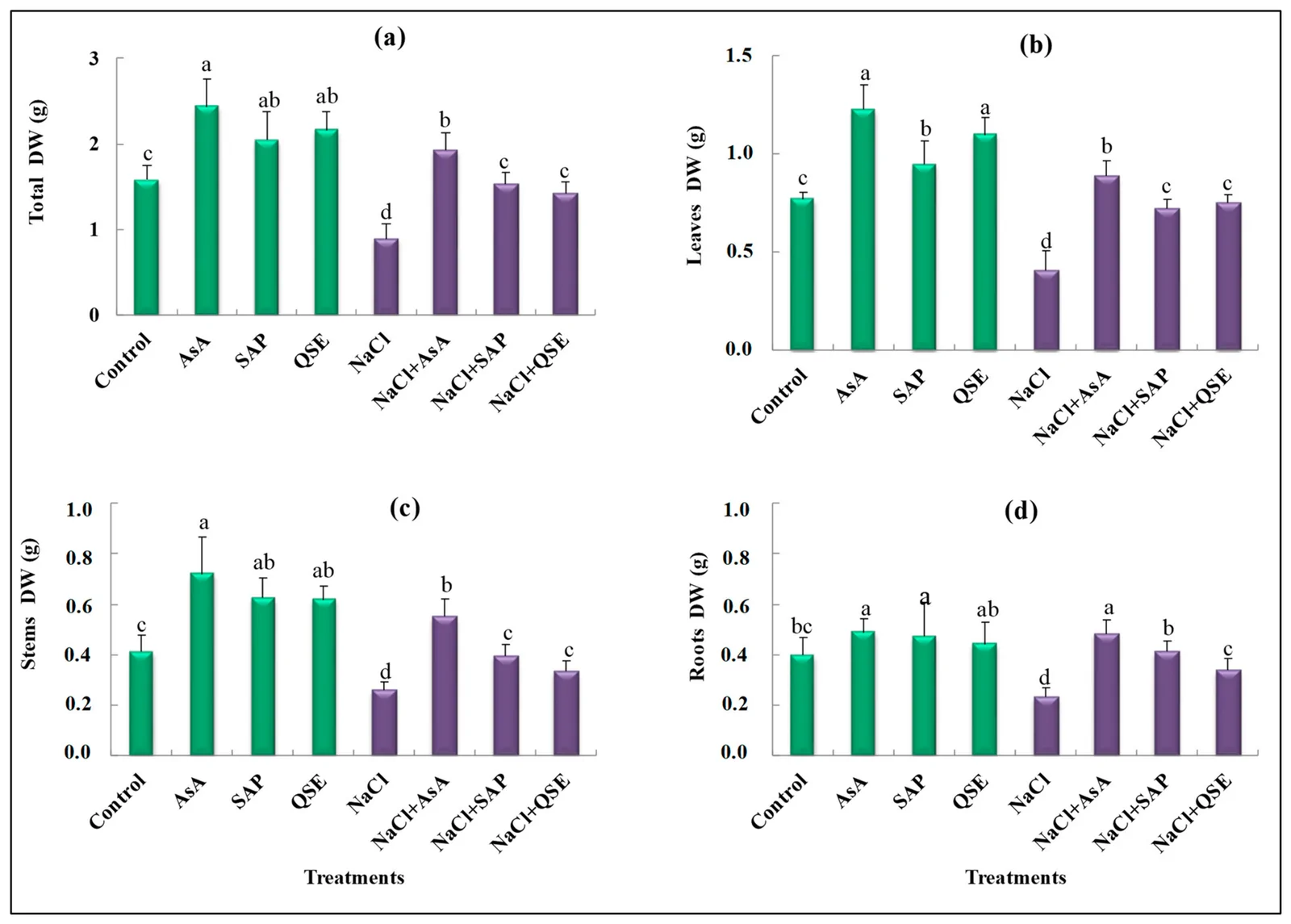
Effects of foliar application of AsA, SAP and QSE on dry biomass, including (**a**) whole plant, (**b**) leaves, (**c**) stems, and (**d**) roots of quinoa plants grown under optimal and saline conditions. Data are presented as means ± SD (*n* = 6). Bars sharing the same letter are not significantly different according to Duncan’s multiple range test at the 95% confidence level.

**Figure 3:**
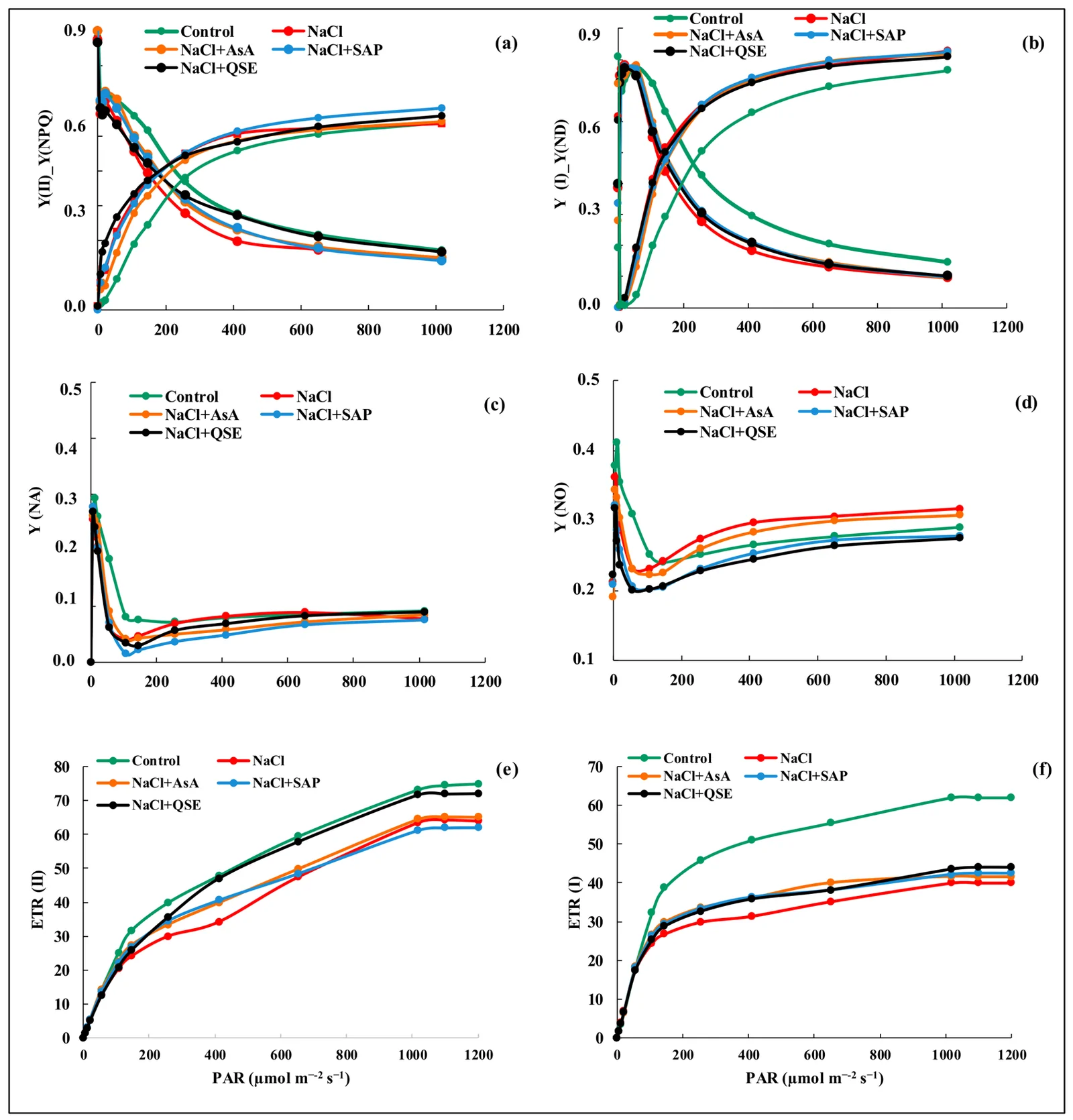
Dual-PAM analysis including: (**a**,**b**) photochemical quantum yield of photosystems I and II; PSI [Y(I)] and PSII [Y(II)]. Quantum yield of non-photochemical energy dissipation in PSII RCs, limited by donor-side Y(ND). Quantum yield of light-induced nonphotochemical fluorescence quenching, Y(NPQ). (**c**) Quantum yield of non-photochemical energy dissipation in reaction centers, limited by acceptor-side Y(NA). (**d**) Quantum yield of non-light-induced non-photochemical fluorescence quenching, Y(NO). (**e**,**f**) Photosystem electron transport rates ETR I and ETR II.

**Figure 4:**
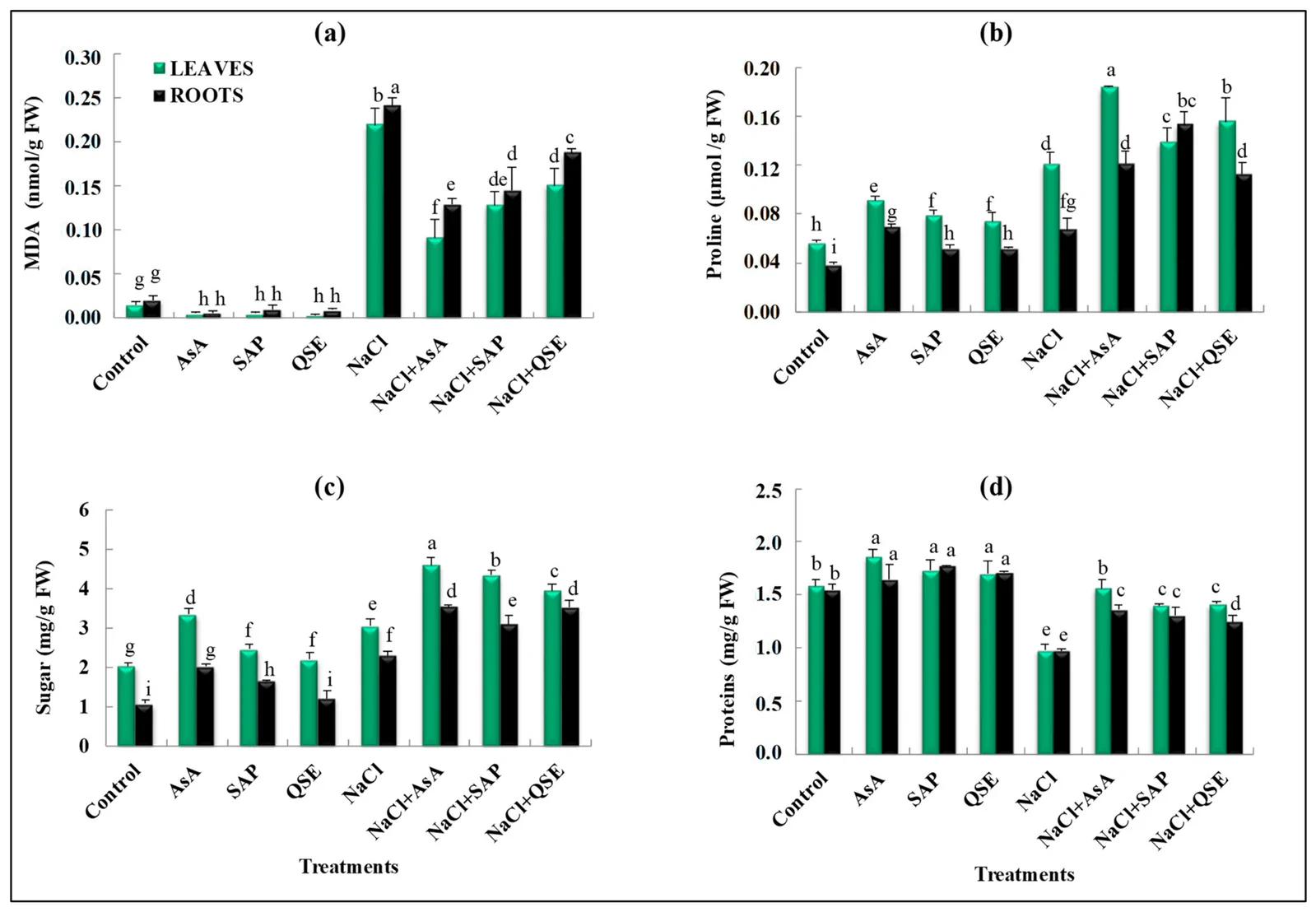
Effects of foliar application of AsA, SAP and QSE on malondialdehyde (**a**), proline (**b**), soluble sugars (**c**) and total soluble proteins (**d**) contents in leaves and roots of quinoa plants grown under optimal and saline conditions. Data are presented as means ± SD (*n* = 6). Bars sharing the same letter are not significantly different according to Duncan’s multiple range test at the 95% confidence level.

**Figure 5:**
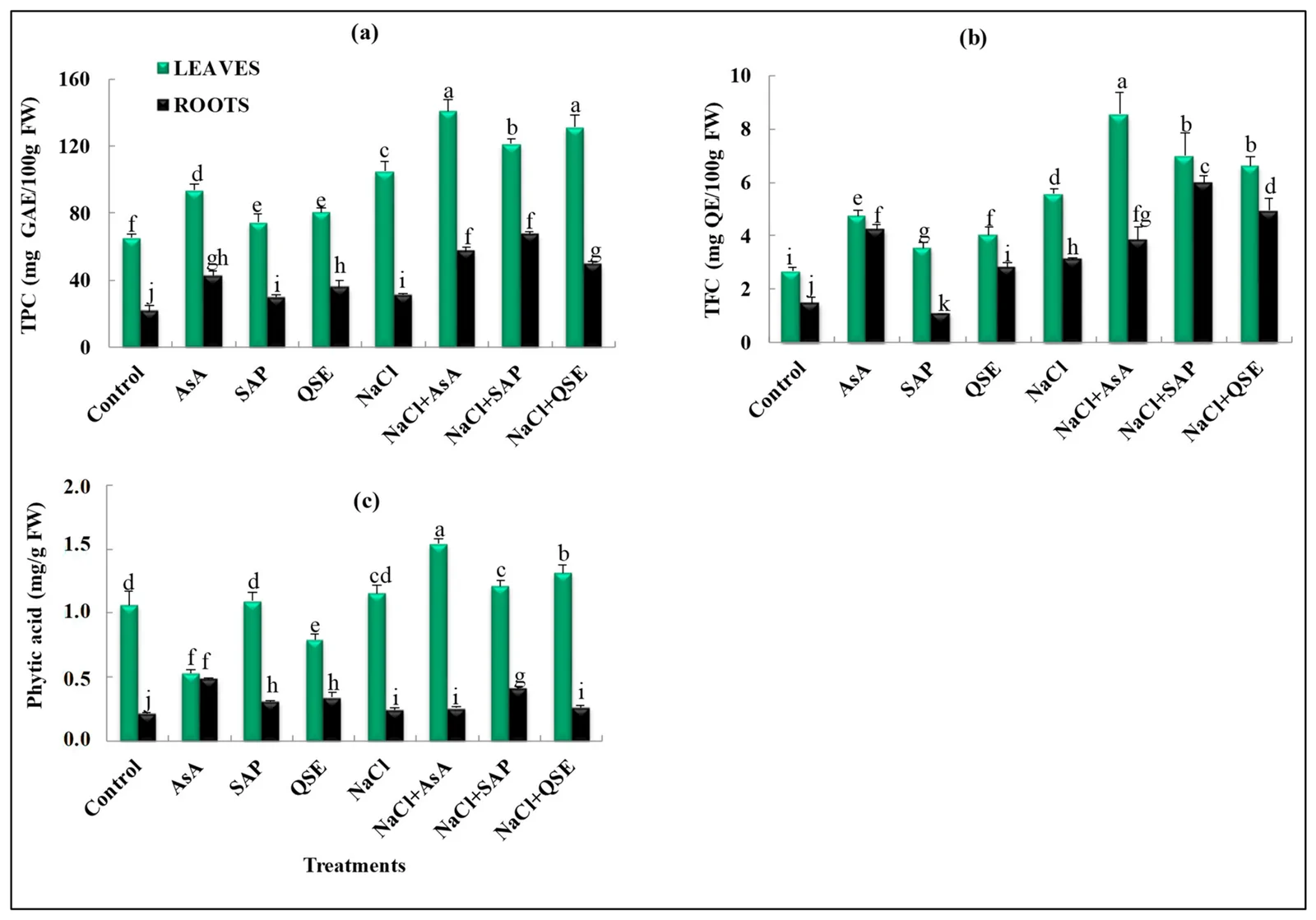
Effects of foliar application of AsA, SAP, and QSE on total phenolic content (TPC) (**a**), total flavonoid content (TFC) (**b**), and phytic acid content (**c**) in leaves and roots of quinoa plants grown under control and saline conditions. Data are presented as means ± SD (*n* = 6). Bars sharing the same letter are not significantly different according to Duncan’s multiple range test at the 95% confidence level.

**Figure 6:**
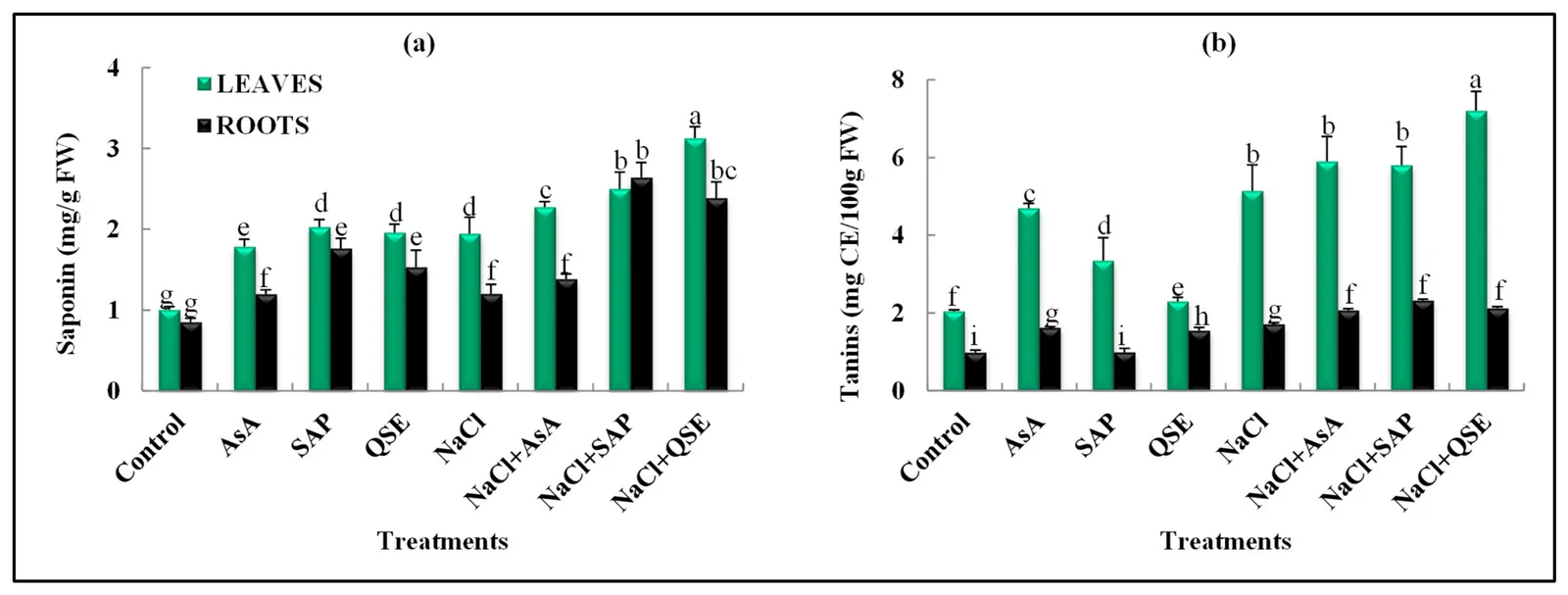
Effects of foliar application of AsA, SAP and QSE on saponin (**a**) and tannin contents (**b**) in leaves and roots of quinoa plants grown under control and saline conditions. Data are presented as means ± SD (*n* = 6). Bars sharing the same letter are not significantly different according to Duncan’s multiple range test at the 95% confidence level.

**Figure 7:**
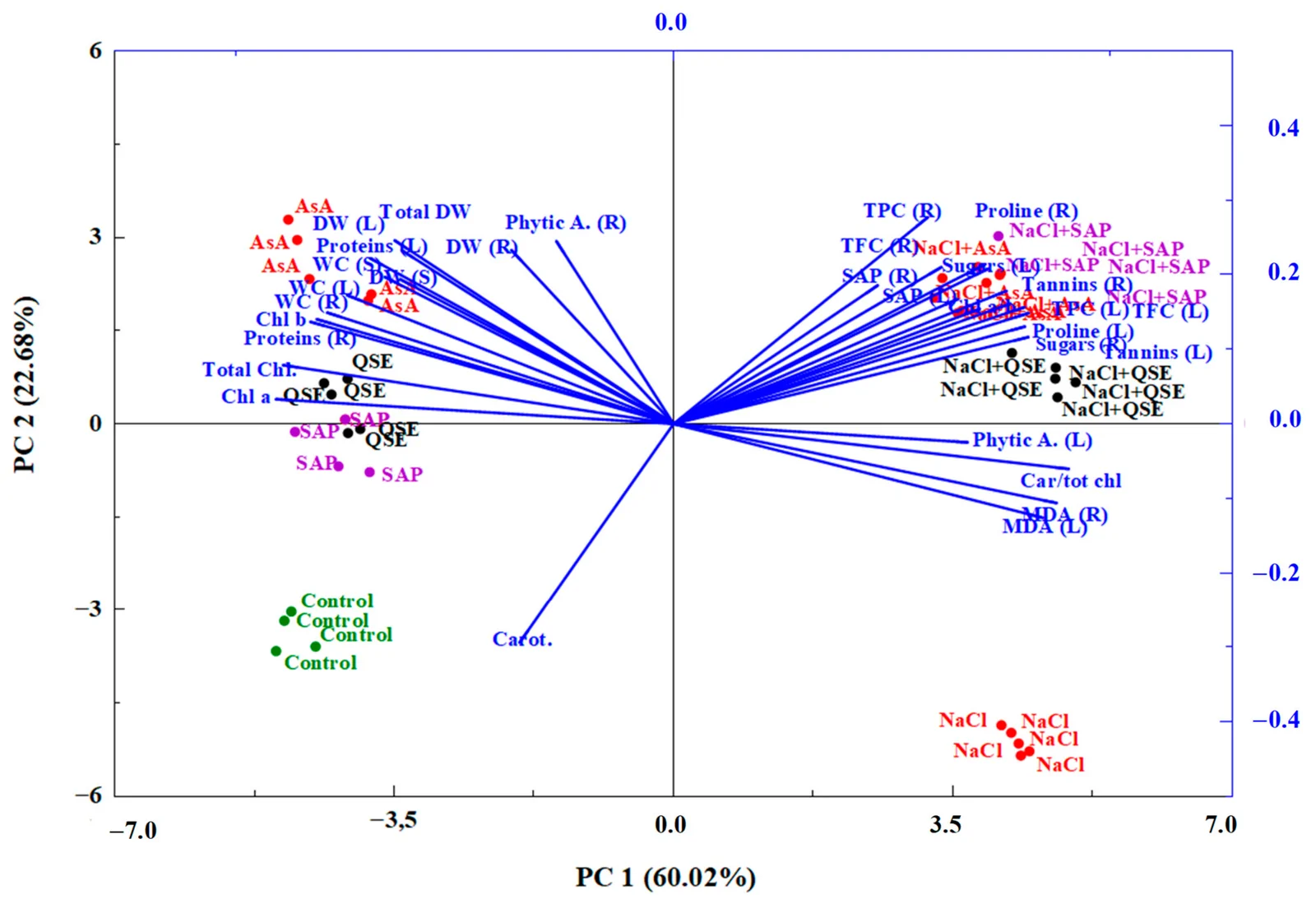
Principal component analysis (PCA) biplot illustrating the relationships among foliar applications of AsA, SAP, and QSE, salinity conditions, and physiological, biochemical, and nutritional traits in quinoa plants. PC1 and PC2 explained 60.02% and 22.68% of the total variance, respectively.

**Figure 8:**
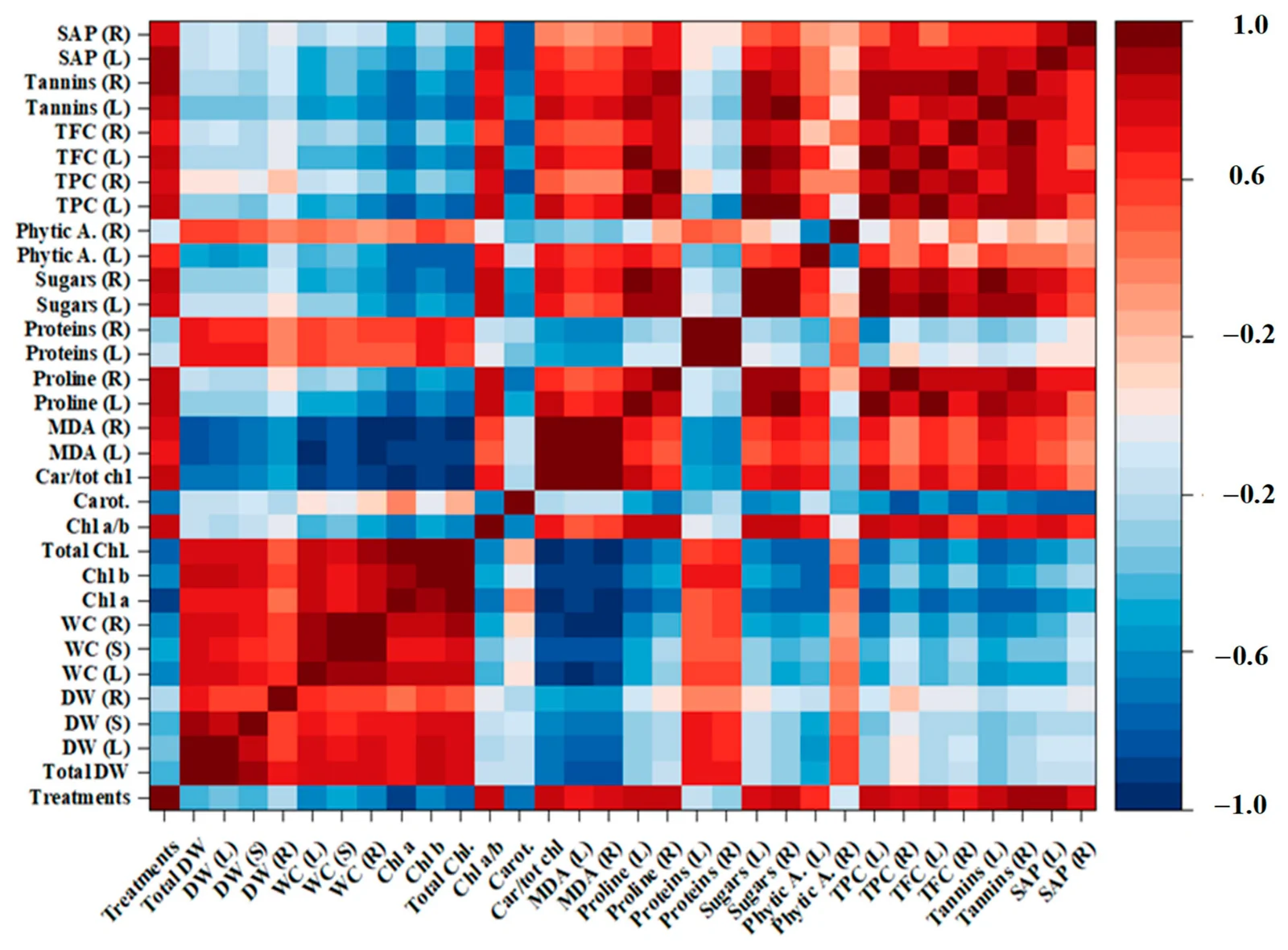
Correlation matrix based on the Pearson correlation coefficient showing relationships among growth, physiological, biochemical, and phytochemical traits in quinoa under different treatments. The color intensity represents the strength of the correlation coefficient, with positive correlations shown in red and negative correlations in blue.

**Figure 9:**
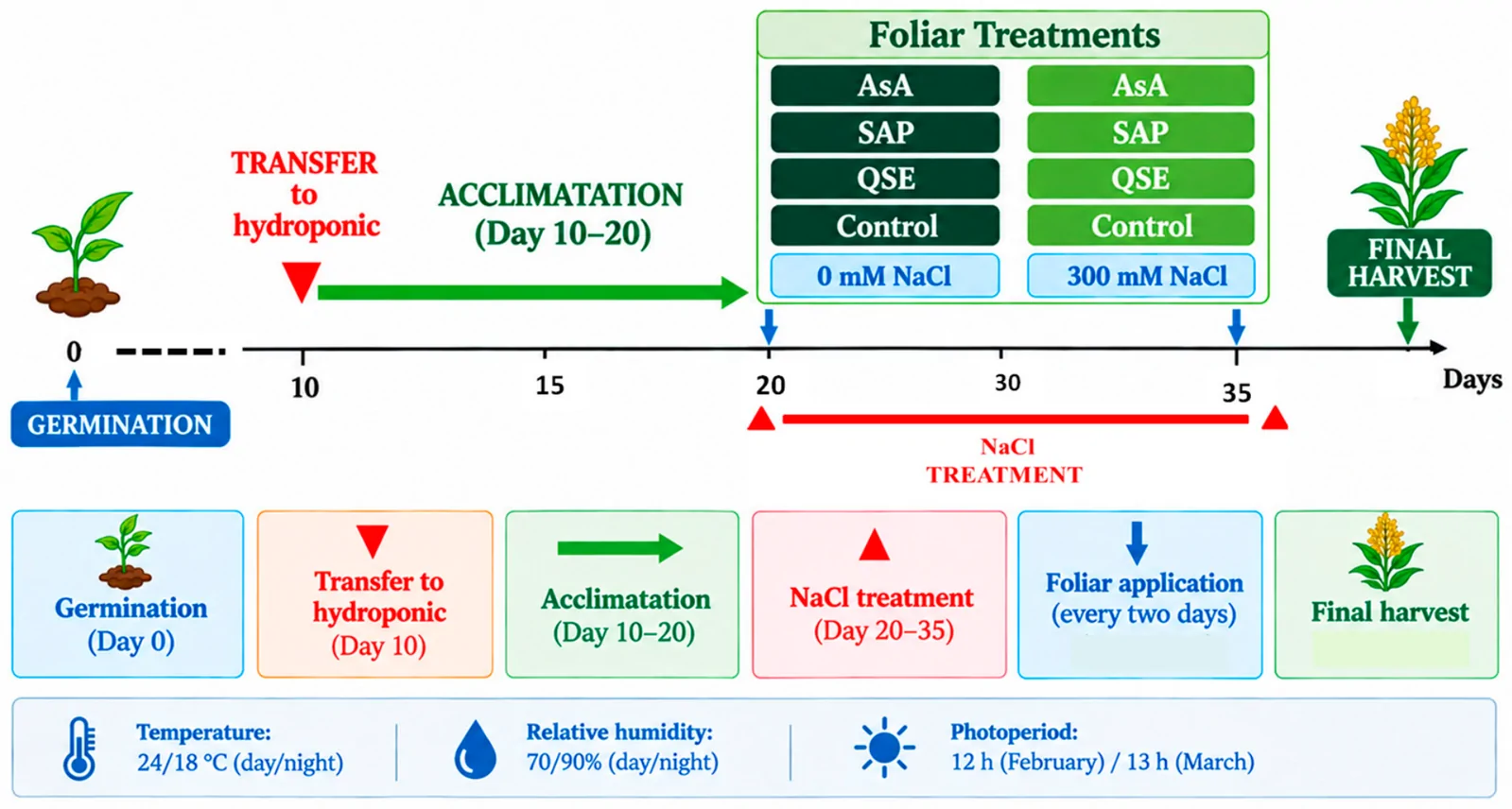
Schematic representation of the experimental design and chronological timeline of treatments and measurements.

**Table 1:** Effects of foliar application of ascorbic acid (AsA), saponin (SAP), and quinoa seed extract (QSE) on the water content (WC) of leaves, stems, and roots of quinoa plants cultivated under control or salinity stress, for two weeks. Data are presented as means ± SD (*n* = 6). Means with similar letters are not significantly different according to Duncan’s multiple range test at the 95% confidence level.

Treatments	Leaves	Stems	Roots
Control	90.94 ± 0.76 *a*	92.82 ± 1.15 *a*	94.12 ± 0.94 *a*
AsA	91.63 ± 0.40 *a*	91.94 ± 1.80 *a*	93.83 ± 1.01 *a*
SAP	90.41 ± 0.98 *a*	91.08 ± 1.41 *a*	93.5 ± 1.85 *a*
QSE	89.12 ± 1.3 *ab*	90.52 ± 1.8 *ab*	93.55 ± 1.79 *a*
NaCl	81.02 ± 0.02 *d*	80.61 ± 1.09 *d*	82.89 ± 1.03 *c*
NaCl + AsA	87.94 ± 0.24 *b*	88.14 ± 1.19 *bc*	88.67 ± 0.38 *b*
NaCl + SAP	87.72 ± 0.83 *b*	89.08 ± 1.61 *b*	89.02 ± 0.09 *b*
NaCl + QSE	84.87 ± 1.74 *c*	87.49 ± 2.82 *c*	88.76 ± 1.03 *b*

Foliar application of QSE also improved plant water status, increasing WC by 5% in leaves, 8% in stems, and 7% in roots compared with the NaCl treatment alone. Among the tested treatments, SAP exhibited the strongest capacity to maintain plant hydration, particularly in stems and roots, followed by AsA, whereas QSE showed a moderate but significant protective effect against salinity-induced water loss.

**Table 2:** Effects of foliar application of AsA, SAP, and QSE on photosynthetic pigments (Chl*a*, Chl*b*, total Chl, carotenoids), Chl*a*/Chl*b,* and Carot/Tot. Chl ratios of quinoa plants cultivated under control or salinity stress for two weeks. Data are presented as means ± SD (*n* = 6). Means with similar letters are not significantly different according to Duncan’s multiple range test at the 95% confidence level.

Treatments	Chl *a* (µg/g FW)	Chl *b* (µg/g FW)	Chl Tot (µg/g FW)	Carot. (µg/g FW)	Chl *a*/*b* Ratio	Carot/Tot. Chl. Ratio
Control	0.65 ± 0.02 *b*	0.37 ± 0.05 *c*	1.02 ± 0.04 *b*	10.13 ± 0.90 *d*	1.77 ± 0.30 *a*	9.91 ± 0.90 *d*
AsA	0.76 ± 0.01 *a*	0.63 ± 0.03 *a*	1.39 ± 0.04 *a*	12.40 ± 1.20 *d*	1.21 ± 0.04 *bc*	8.87 ± 0.70 *d*
SAP	0.69 ± 0.02 *ab*	0.51 ± 0.06 *b*	1.20 ± 0.05 *ab*	16.13 ± 1.40 *c*	1.37 ± 0.19 *b*	13.35 ± 0.98 *d*
QSE	0.61 ± 0.03 *b*	0.55 ± 0.04 *ab*	1.16 ± 0.04 *ab*	13.55 ± 1.8 *cd*	1.11 ± 0.12 c	11.61 ± 1.23 *d*
NaCl	0.16 ± 0.01 *e*	0.10 ± 0.005 *e*	0.26 ± 0.01 *d*	15.48 ± 0.67 *c*	1.59 ± 0.08 *ab*	57.16 ± 2.55 *a*
NaCl + AsA	0.24 ± 0.01 *c*	0.21 ± 0.026 *d*	0.45 ± 0.018 *c*	21.58 ± 0.59 *a*	1.14 ± 0.16 *bc*	46.93 ± 1.51 *b*
NaCl + SAP	0.20 ± 0.02 *d*	0.26 ± 0.024 *d*	0.46 ± 0.016 *c*	19.72 ± 1.68 *ab*	0.76 ± 0.12 *d*	42.19 ± 3.52 *c*
NaCl + QSE	0.18 ± 0.01 *de*	0.20 ± 0.005 *d*	0.38 ± 0.009 *c*	18.56 ± 1.25 *b*	0.88 ± 0.04 *d*	46.97 ± 4.20 *b*

**Table 3:** Experimental treatments applied to quinoa plants under control and saline conditions. (+) Indicates presence and (−) absence of the considered solution. AsA: ascorbic acid; SAP: Saponins; QSE: Quinoa seed extract.

Treatment	NaCl 300 mM	Foliar Spray AsA (0.2 mM)	Foliar Spray SAP (0.15%)	Foliar Spray QSE (3%)
Control	−	−	−	−
AsA	−	+	−	−
SAP	−	−	+	−
QSE	−	−	−	+
NaCl	+	−	−	−
NaCl + AsA	+	+	−	−
NaCl + SAP	+	−	+	−
NaCl + QSE	+	−	−	+

## Data Availability

The data that support the findings of this study are available from the corresponding author upon reasonable request.

## Abbreviations

The following abbreviations are used in this manuscript:

AsA	Ascorbic Acid
BBCH	Biologische Bundesanstalt, Bundessortenamt und CHemische Industrie scale
DW	Dry weight
FW	Fresh weight
MDA	Malondialdehyde
PSI	Photosystem I
PSII	Photosystem II
QSE	Quinoa Seed Extract
ROS	Reactive Oxygen Species
SAP	Saponins
TFC	Total Flavonoid Content
TPC	Total Phenolic Content
WC	Water content
